# Acquisition of Pitjantjatjara Clause Chains

**DOI:** 10.3389/fpsyg.2020.00541

**Published:** 2020-04-16

**Authors:** Rebecca Defina

**Affiliations:** Research Unit for Indigenous Language, ARC Centre of Excellence for the Dynamics of Language, The University of Melbourne, Melbourne, VIC, Australia

**Keywords:** Pitjantjatjara, language acquisition, clause chain, clause combining, converb, Australian languages

## Abstract

In Pitjantjatjara, a central Australian Indigenous language, speakers typically describe sequences of actions using clause chaining constructions. While similar constructions are common among the world’s languages, very little is known about how children acquire them. A notable exception are the converb constructions of Turkish, which have been relatively well-studied. The present paper examines the acquisition of Pitjantjatara clause chaining constructions and compares this with the acquisition of Turkish converb constructions. Data is drawn from a naturalistic corpus recorded between 2016 and 2019. The corpus contains over 4000 utterances from 23 children aged between 10 months and 10 years, five of whom are recorded at multiple ages. The corpus also includes approximately 1600 utterances from 21 adults, aged between 16 and 70. Results show that the acquisition of Pitjantjatjara clause chains consists of three stages. Stage 1 features juxtaposition of finite verb forms. In Stage 2, children make regular use of clause chain morphology, but primarily for modification purposes. In Stage 3, clause chains are the preferred strategy for sequential actions as well as modification purposes. The initial use of verb juxtaposition followed by increasing use of dedicated morphology is consistent with findings for Turkish converb acquisition, with speakers of both languages utilizing dedicated forms from around 2;6 onwards. A notable difference between the acquisition of Pitjantjatjara clause chains and Turkish converbs is in the order of acquisition of semantic functions. In Turkish, children acquire temporal functions, such as sequential actions, before modifying functions, such as manner specification. In Pitjantjatjara, the order is reversed, with children first using clause chaining constructions for modification and simultaneous aspects of events before utilizing them to combine sequential actions. This raises questions regarding the distribution and relative timing of event combination and modification strategies.

## Introduction

Speakers of the Australian Indigenous language Pitjantjatjara often describe connected sequences of actions using clause chains (Goddard, 1988). These clause chain constructions consist of one or more non-finite verbs and one finite verb inflected for tense, aspect, or mood. The finite verb is typically chain final. The non-finite verbs are marked with a distinctive clause chain suffix and their tense, aspect, and mood values are inferred from those of the finite verb. A classic example can be seen in (1), with verbs marked in bold.


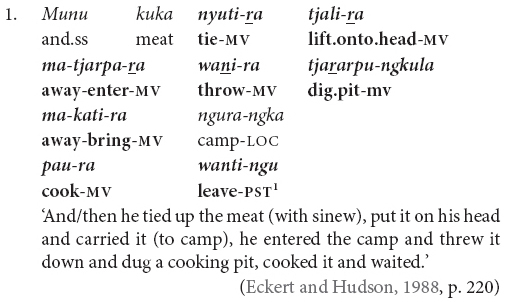


Pitjantjatjara clause chaining constructions bear similarities to the clause chaining constructions of Papuan languages such as Nungon (Sarvasy, 2015) and Yimas (Foley, 1991, 2010). They also resemble the converb constructions of languages like Turkish (Slobin, 1995) and Japanese (Alpatov and Podlesskaya, 1995). They do not resemble the similarly named coverb constructions frequently reported in northern Australia. Coverbs refer to a distinct word class used in combination with verbs to create complex predicates (Baker and Harvey, 2010). Converb and clause chaining constructions are not widespread among Australian languages.

In this paper, I follow Haspelmath (1995) in viewing syntactic subordination as the distinguishing characteristic of converb as opposed to clause chaining constructions. Since the Pitjantjatjara constructions do not typically involve syntactic subordination – as discussed below – they are best viewed as clause chaining constructions. I then follow the Papuan terminology in referring to the non-finite verbs in the clause chain as *medial*.

Unfortunately, vanishingly little is known about the acquisition of converb or clause chaining constructions crosslinguistically. The notable exception to this is Turkish, which has been the focus of several studies (e.g., Slobin, 1982, 1995; Aksu-Koç and Slobin, 1985; Çapan, 2013). As Haspelmath (1995) notes, converb and clause chaining constructions share many common properties and are largely comparable. These strong similarities and the current data limitations motivate the present comparison between Pitjantjatjara clause chains and Turkish converbs.

The research on Turkish has found that children first juxtapose verbs, without converb morphology, in order to achieve converb-like functions (Aksu-Koç and Slobin, 1985). Then, from around the age of 2;6 onwards, Turkish speaking children begin to use converb morphology, as well as conjunctions, for expressing temporal and causal sequences (Aksu-Koç and Slobin, 1985). Temporal uses remain dominant until around 5 years of age, when children begin to use converbs for a broader range of semantic functions including manner modification (Çapan, 2013). Finally, Slobin (1995) reports that the use of the converbs to combine elements into a single composite event, rather than a sequence of distinct events, is not acquired until approximately 7 years of age.

The present paper provides an initial account of how children acquire Pitjantjatjara clause chain constructions and to what extent this resembles the patterns observed in the acquisition of Turkish converbs. Before turning to the details of the study it is necessary to introduce the Pitjantjatjara language and its clause chains in more detail.

## Background

### Introduction to Pitjantjatjara

Before colonization, 250 or more Indigenous languages were spoken throughout Australia. Today, approximately 120 of these are still spoken, with only 13 being learnt by children (Marmion et al., 2014). Pitjantjatjara (Glottocode: pitj1243, ISO 639-3: pjt) is one of these few Indigenous Australian languages still being learnt as a first language. It is currently spoken by roughly 3000^2^ people, living primarily around the tristate region where the states of Western Australia and South Australia meet the Northern Territory, in the desert region of central Australia.

The language is classified as part of the Wati-Nyungic branch of Pama-Nyungan (Bowern and Atkinson, 2012) and forms part of the Western Desert dialect chain which extends across much of central and western Australia. Pitjantjatjara and its closest sister dialect, Yankunytjatjara, are largely mutually intelligible and are both described in one grammar (Goddard, 1985) and dictionary (Goddard, 1996).

Pitjantjatjara has relatively free word order, although the order Subject Object Verb has been reported as occurring more frequently (Bowe, 1990). Grammatical relations are indicated by case marking according to a tripartite case system common amongst Australian languages (Goddard, 1982). The three core cases, nominative (subject of intransitive verb), ergative (subject of transitive verb), and accusative (object of transitive verb), are marked differently for pronouns, common and proper nouns. There is a split case-marking system, where pronouns are marked according to a nominative-accusative pattern and other nominals are marked according to an ergative-absolutive pattern.

Verbs are divided into four conjugation classes with distinct inflectional paradigms (Goddard, 1985). Verb class membership is largely based on phonology (specifically the number of morae) and transitivity. The two major conjugation classes consist primarily of verbs with an even number of morae and are either predominantly intransitive (class -Ø), e.g., *nyina* ‘sit’ and *pitja* ‘come,’ or transitive (class -l), e.g., *mantji-l* ‘get’ and *ngalku-l* ‘eat.’ The other classes (-n and -ng) consist of verbs with an odd number of morae, e.g., *tju-n* ‘put’ and *nya-ng* ‘see.’ Both these classes contain many transitive verbs, though the -ng class is used for intransitives derived with the inchoative -*ri*, e.g., *paku-ri-ng* ‘become tired.’ Finite verbs are inflected for tense, aspect, and mood with the following distinctions: past (perfective or imperfective), present, future, habitual, imperative (perfective or imperfective) (Goddard, 1985).

### Pitjantjatjara Clause Chains

Pitjantjatjara employs a clause chain construction in which a string of one or more non-finite verbs occurs with one finite verb. The chain typically describes sequential or simultaneous actions. All verbs typically share a single subject. Transitive verbs can, but do not necessarily, share objects. All verbs in the chain are understood to share the same broad temporal frame, made explicit in the tense, aspect, mood marking of the finite verb. The finite verb may also be nominalized in which case it does not carry tense, aspect, mood information. The most frequent example of this is in the negative, which is a nominal category in Pitjantjatjara and requires verbal nominalization. The form of the non-finite medial verb suffix varies only according to the conjugation class of the verb root. The finite verb is typically chain-final, as in (1) and (2), but not always, as in (3) and (4). Chains commonly consist of only two verbs, as in examples (2–4), but longer chains are often reported, for instance the eight verbs in example (1) above.


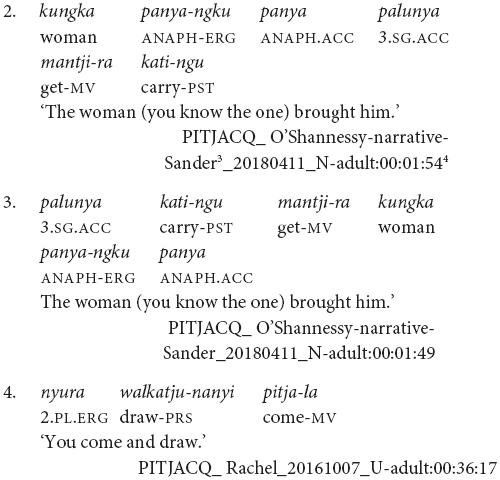


The verbs in Pitjantjatjara clause chains typically share a subject, as in (1–4). This co-reference is typically full co-reference but may at times be partial, as shown by Goddard (1988) for Pitjantjatjara’s sister dialect Yankunytjatjara. In (5), the subject of the first verb is included in the subject of the second, and in (6) we see the reverse, where the subject of the second verb is a subset of the subject of the first verb.


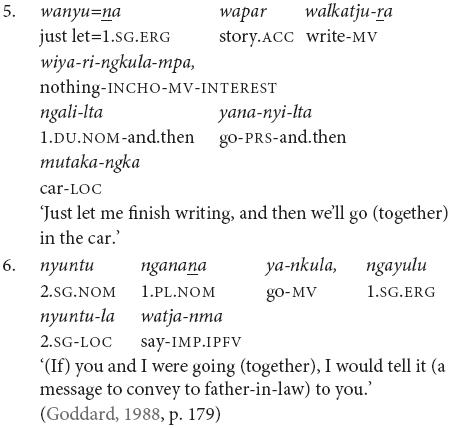


It is also possible for the verbs to have distinct subjects. Subject switch in Pitjantjatjara clause chains is typically marked with *ka*, as in example (7). *Ka* is also used to coordinate clauses with distinct subjects, as in (8). I have observed that speakers do not always mark the switch of subject when the context is sufficiently clear, for example see (9) and (10). At present, the only examples of unmarked subject switch I have observed from adults occur in narratives and with verbs which Goddard (1985, p. 106) described as denoting an “ambient change,” for example (10). It is possible that unmarked switch-subject is a feature of child, rather than adult, Pitjantjatjara clause chaining.


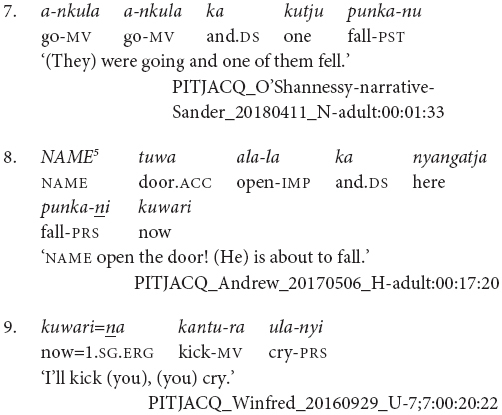



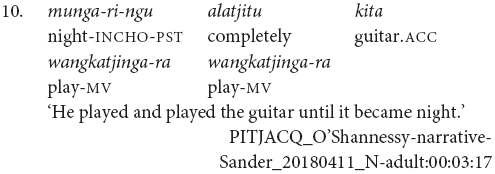


The order of verbs does not necessarily match iconic event order. This can be seen in (3), (4), and (10), where the action referred to by the finite verb occurs after that referred to by the medial verb, even though the finite verb precedes the medial verb. There is a strong tendency for the action described by the finite verb to temporally follow or overlap with actions described by medial verbs. This is not a strict rule, however, as can be seen for instance in example (25). The pattern of chain-final medial verbs with non-iconic event ordering resembles the postposed medial clauses of Nungon (Sarvasy, 2015).

The examples in (2) and (3) show some of the word order variations possible in Pitjantjatjara. Here we have the same speaker referring to the same event in consecutive utterances first with the order Object Verb_FINITE_ Verb_MEDIAL_ Subject, in example (3), and then Subject Object Verb_CONVERB_ Verb_FINITE_, in example (2). This second order is possibly more common, but the first ordering is equally acceptable. According to Stassen (1985) and Givón (1990), finite-final clause chains are typologically associated with Object-Verb basic word order whereas finite-initial clause chains are typologically associated with Verb-Object basic word order. Since Pitjantjatjara word order is free, it would be plausible for the position of the finite verb to also vary freely. However, this is not the case. Bowe (1990) presents evidence suggesting that the position of the finite verb reflects two distinct syntactic subtypes. When the chain is finite-final, as is most frequent, the case of the shared subject is determined by the chain as a whole – if any verb in the chain is transitive, the subject is marked ergative, as in (11) and (12). However, if the chain is finite-initial, the case of the subject is determined by the finite verb alone. This can be seen in (13) where the subject is nominative if the intransitive verb is finite, but ergative if the transitive verb is finite.


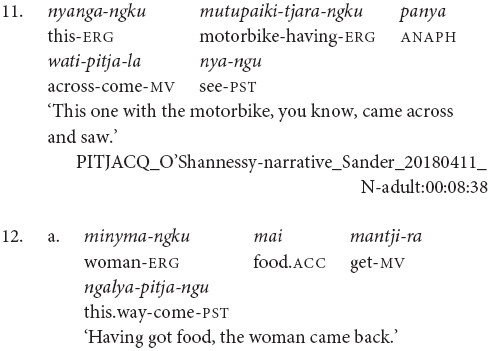



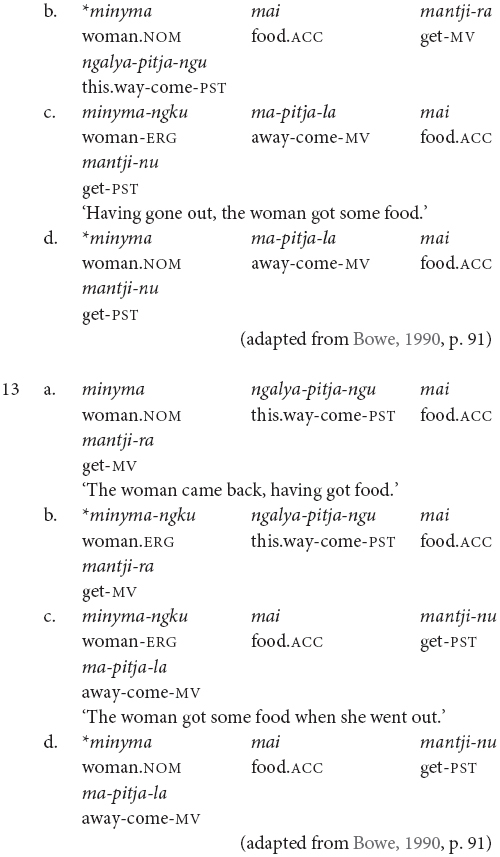


These case marking patterns suggest that the finite-initial chains involve syntactic subordination while the finite-final chains do not. In the finite-initial cases, the transitivity of the medial verb does not impact on the case marking of the matrix clause subject. This indicates that the medial verb phrase is syntactically subordinate to the matrix clause. In contrast, with the finite-final chains, the transitivity properties of both verbs impact on the case marking of the subject, indicating that neither is syntactically subordinate.

Pitjantjatjara clause chains may also be divided according to their clausality. Goddard (1985, 1988) distinguishes these as Tight versus Loose constructions. The Loose constructions are those such as (1), (5), (6), (7), and (11). They can contain two or more verbs; arguments and other elements may occur between the verbs; and the verbs may have distinct or shared arguments. These are multiclausal constructions – chains of clauses. In contrast, Goddard’s Tight constructions are like those in (2), (3), and (4). These constructions contain only two verbs. The arguments are shared and are expressed to either side of the verbs, with no elements occurring between the verbs. The verbs refer to actions with a tight semantic link and work together to form a single predicate within a single clause. While this distinction is important for the syntactic description of Pitjantjatjara clause chains, it is often difficult, or impossible, to determine in practice whether a particular example consists of multiple clauses or not. Most Pitjantjatjara clause chains consist of two verbs only and, given argument ellipsis and word order variations, the two verbs are often adjacent. In these cases, Goddard (1985, 1988) relies on the typicality of the verb pairing. There is, however, evidence to suggest that individual verb pairings can occur in both multi- and monoclausal chains. For instance, the verbs ‘go’ and ‘gather’ are identified by Goddard (1985, 1988) as a typical Tight construction verb pair, as seen in (14). However, these same verbs frequently occur in my data with the object intervening and therefore as a Loose construction, as seen in (15). Given the difficulties in consistently distinguishing multiclausal and monoclausal chains, I will not attempt to make the distinction in the present analysis.


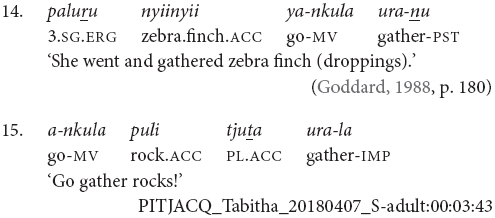


While Pitjantjatjara clause chains are predominantly used to refer to sequences of actions, as described above, some Pitjantjatjara clause chains can be used for modificational, rather than sequential, functions. Possibly the most common of these is the repetition of a verb in the medial form to indicate that the event was repeated or extended over a long time. The number of repetitions indicates the degree of repetition or extension. The example in (16) shows one of these repetitions within a sequential action clause chain.


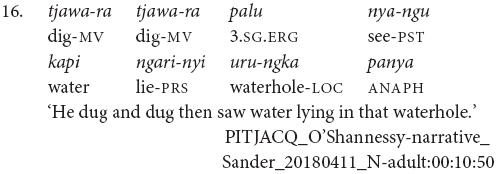


A subset of verbs, such as *wiru* ‘make beautiful’ and *alatji* ‘do like this,’ can be used as adverbials in their medial form. In these cases, the finite verb is semantically and syntactically dominant and determines the case marking of the subject, as can be seen in example (17).


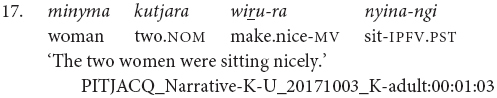


Conversely, there is a set of verbs which can be used as finite verbs in clause chains with an aspectual function. For instance, in example (18), it is the intransitive medial verb *ngara* ‘stand’ which controls the nominative case marking of the subject, while the transitive finite verb *wani* ‘throw’ indicates that the action, or stance in this case, is distributed. Note that these medial and finite modifying verbs are often semantically bleached.


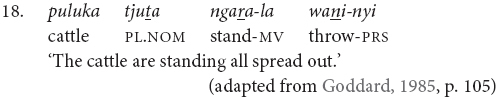


Finally, while clause chains are a preferred way to describe sequential actions in Pitjantjatjara, there are alternatives: clause juxtaposition; coordination; and subordinate circumstantial clauses. Clause juxtaposition and coordination involve sequences utterances each with phrase final intonation patterns, marked here by a comma, as can be seen in the excerpt of a narrative provided in (19). The subordinate circumstantial constructions combine a maximum of two actions and make a same- versus different-subject distinction. The different-subject form -*nyangka* can be seen in example (20a) and the same-subject form -*nytjatjanu* can be seen in (20b). The same-subject form is more semantically restricted and can only be used when there is a strictly sequential temporal relationship between the actions, in other cases a clause chain is used. The order between subordinate and finite clauses varies and does not necessarily relate to iconic event order, as can be seen in examples (21) and (22). Bowe (1990) convincingly argues for the subordinate status of these clauses based in part on the fact that they do not influence the case marking of the matrix clause subject, as can be seen in example (23).


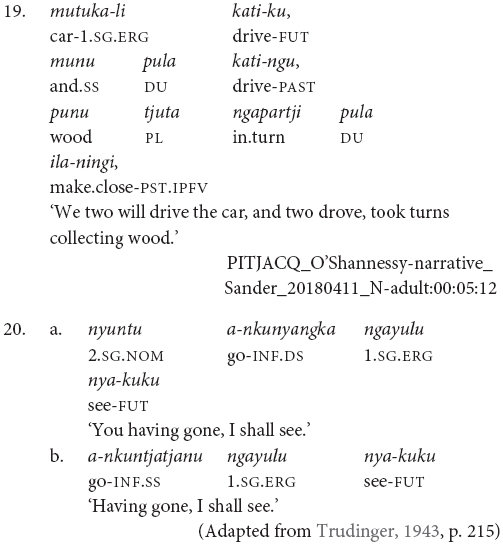



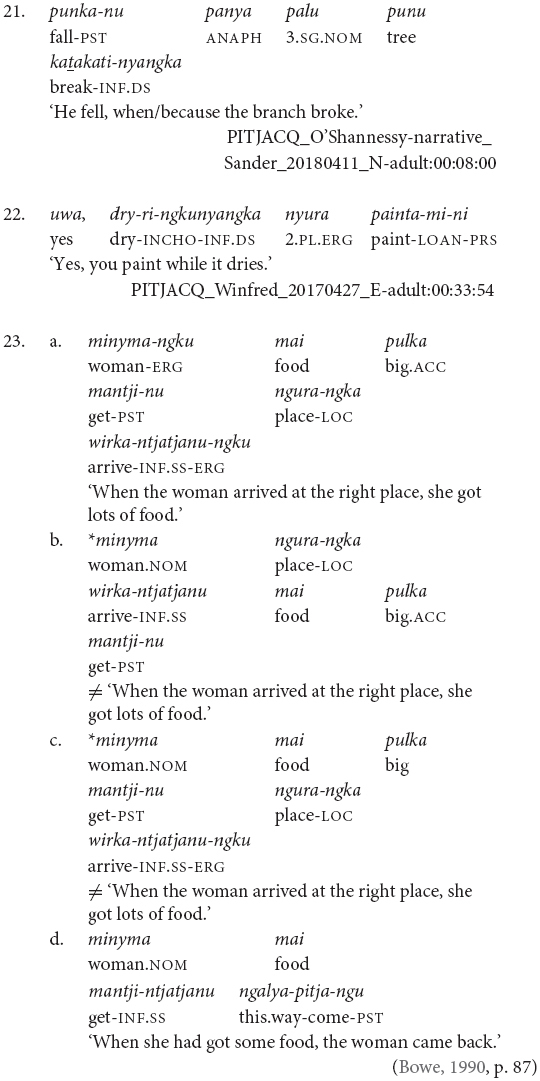


Pitjantjatjara clause chains and related constructions are summarized above in Table 1. There are many similarities with Turkish converbs and related constructions, but also some differences. Both languages employ chains of non-finite verbs together with a finite verb form to combine sequential and simultaneous actions, as well as using non-finite for more modifying, adverbial-type functions. However, while Pitjantjatjara has a single medial form, which is used with all converb functions, Turkish has a range of converb forms, a more generic form -*ip* resembling the Pitjantjatjara medial verb form as well as several others with more specific semantics (Slobin, 1995). Pitjantjatjara clause chains are predominantly used in same-subject situations, where there is a single, shared subject for all verbs in the construction. In contrast, individual Turkish converb markers can either be same-subject, different-subject, or used in both contexts (Slobin, 1995). Turkish also makes a morphological distinction between converbs which are specialized for ‘looser’ temporal linkage, such as -*ince* and *-erken*, and a converb form *-erek*, which is used to bind elements together into a single composite event (Slobin, 1995). Both languages also employ nominalized verbs in subordinate clauses as alternative strategies for linking sequential and simultaneous actions.

**TABLE 1:** Overview of Pitjantjatjara clause chains and related constructions.

Clause chain					Subordinate circumstantial	

Sequential				Modifying	Switch subject	Same subject
Finite-initial	Finite-final	Loose	Tight		-*nyangka*	-*nytjatjanu*
					
		**(Distinction collapsed for present study)**				
Finite verb controls case marking Medial verb clause is subordinate	All verbs influence case marking	One or more medial verbs Arguments and other elements can occur between the verbs Loose connection between actions	No more than one medial verb No arguments between verbs Tight connection between actions	Adverbial, postural, aspectual modification Finite verb plus maximally one lexically distinct medial verb	Temporal sequence or overlap, as well as causal interpretations Main clause plus maximally one subordinate clause	Temporal sequence only Main clause plus maximally one subordinate clause

If Pitjantjatjara children acquire these constructions in a similar way to Turkish children, we might expect: an initial stage of verb juxtaposition before use of dedicated morphology; use of clause chain based strategies before subordinate strategies; and the use of clause chains for loose temporal linkage before adverbial modification and finally tighter binding of sequential elements into a single event.

## Materials and Methods

### Participants

The present research is based on data collected in Pukatja (also known as Ernabella), one of the largest communities in the Anangu Pitjantjatjara Yankunytjatjara (APY) lands of central Australia and home to approximately 500 people. Pitjantjatjara is the primary language of the community and the first language of the majority of children, some of whom also speak other languages, including Warlpiri and Yolngu Matha, in the home. All children subsequently learn English at school, typically from 3 years of age onwards.

**FIGURE 1:**
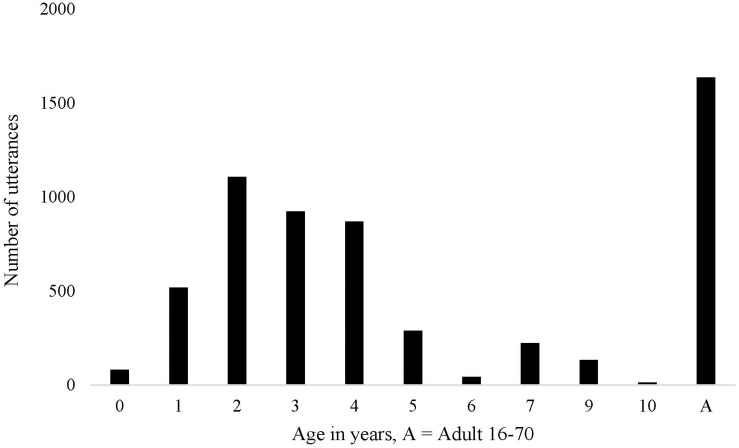
Number of utterances per age group.

The data for this study comes from an ongoing longitudinal investigation of Pitjantjatjara language acquisition. This broader study includes 13 focus children aged between 10 months and 4 years at time of first recording. Ideally, focus children are recorded at least once every 6 months over 3 years, however, the tendency for families to travel means there are many missing timepoints in this dataset. The ongoing process of transcription means there are yet more gaps in the currently available dataset. For the purposes of this study, I include all currently transcribed speech from the corpus. This includes approximately 4200 utterances from 28 children aged 10 months to 10 years and 1600 utterances from 21 female adults between the ages of 16 and 70, see Figure 1 for an overview of the data distribution across the ages. The majority of the child utterances (67%) are produced by five focus children: Simon (age range 1;4–2;11^6^), Andrew (1;9–4;3), Daniel (2;8–5;3), Rachel (2;11–3;6), and Emily (2;11–3;11). The other 23 children have either been transcribed in one session only or are non-focus children who appear in the recordings. Given the current gaps in the longitudinal data, I first analyze the data cross-sectionally, including the speech of all children at similar ages and stages of development. Finally, I check that the cross-sectional findings are consistent with the individual trajectories of these five focus children.

### Data Collection

Data was recorded in naturalistic settings with the focus child freely interacting with one or more adult caregivers and, in most cases, other children as well. Individual recording sessions range between 30 min and 3 h in duration. The focus children wore a small bag with a microphone which recorded their own speech, as well as that of others nearby. Most sessions were also video recorded. Adults and children were aware that the focus of the activity was the child’s speech and in some cases adults encouraged children to talk, at other times adults left the children free to determine how they engaged with the recording session, including how much, or whether, they talked, what they talked about, and what activities they engaged with.

### Data Analysis

Recordings were transcribed in ELAN (Wittenberg et al., 2009) with the assistance of Pitjantjatjara native speakers, typically the mother or grandmother of the focus child. Utterance boundaries were determined by conversational turns or intonational breaks. Child utterances were transcribed phonologically and, where it differed, the caregiver’s interpretation was transcribed on a separate tier.

Instances of clause chains, subordinate circumstantial constructions, and any other utterances with multiple verbs within a single intonation unit were noted and counted. Repetitions of a recent utterance by the child or an interlocutor were not included in the token counts. Clause chains were coded for the number of distinct lexical verbs, finite verb position, and function. The mean length of utterance (MLU) was calculated if the speaker produced 50 utterances or more within the recording session. It was calculated as a morpheme count per utterance, excluding false starts and repetitions (but counting functional reduplication), and counting irregular or portmanteau forms as single morphemes. All statistical analyses were performed using R (R Core Team, 2018).

## Results and Discussion

### Adult Usage

Before considering the trajectory of acquisition, it is helpful to examine how adults use clause chains in their speech to and around children. The corpus contains 1637 utterances from 21 adults, all female. These adults are predominately mothers, grandmothers, and aunts of the focus children. Transcription efforts have focused on child-directed speech, so these utterances are largely child-directed.

An initial examination of utterances according to the age of addressee was performed. The mean length of utterance (MLU) was reduced with younger addressees, as is common with child-directed speech (e.g., Cross, 1977). This reduction was particularly noticeable in speech to infants. The average MLU of all adult utterances directed to the youngest child (0;10) was 1.5 morphemes per utterance. In contrast, the MLU of adult speech directed to 3- and 4-year-olds, 3.3, approached that of speech directed to adults, 3.7. Reduction of the average utterance length was most commonly achieved by ellipsis of arguments (e.g., *punkanu* ‘fell’ for ‘you fell’) or frequent utterances consisting of a single nominal (e.g., *tjutju* ‘doggiewoggie’). No differences in the use of clause chains were observed according to age of addressee: The frequency of use, length of the chain, and morphosyntactic and semantic types of clause chains were all similar, regardless of the age of the addressee. This does not necessarily mean that adult Pitjantjatjara speakers do not modulate their clause chain use according to age of addressee, but a more thorough investigation of both adult- and child-directed speech would be needed to evaluate this.

Clause chains were present in 56 of the 1607 utterances, i.e., 3%, as seen in Table 2. This frequency is much lower than suggested by earlier descriptions of the language (Goddard, 1985; Bowe, 1990), a point I will return to below.

**TABLE 2:** Adult clause chain use: number of tokens of each construction type.

Juxtaposed finite		Clause chain			Subordinate	
Sequential	Simultaneous	Sequential	Simultaneous	Medial only	Switch subject	Same subject
12	1	31	21	4	7	0

Thirty-one of the clause chains described sequential actions, as in examples (24 and 25). Twenty were used for adverbial or aspectual modification, as in examples (26 and 27).


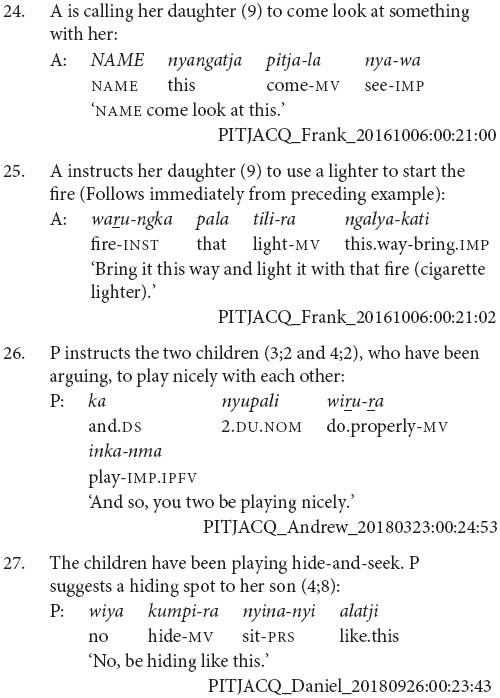


The majority of the clause chain constructions are finite-final. However, 12 or 21% are finite-initial, as in examples (28 and 29).


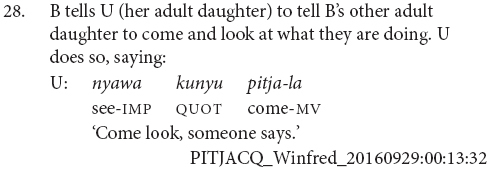



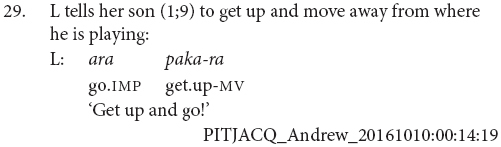


Most chains consisted of two verbs, and the longest chains contained three verbs, as in example (30). The average number of verbs in a chain was 2.1. There were also sentences with medial verbs but no finite verb, as in example (31). These medial-verb-only clauses were not reported in previous descriptions of the language. They are used for polite imperatives or continuing aspect, functions which are also noted for similar non-canonical medial verb clauses in the Papuan language Nungon (Sarvasy, 2015).


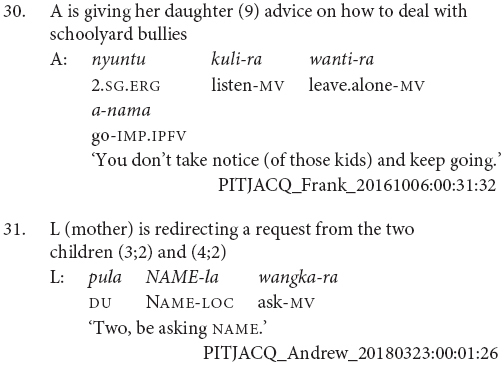


There were no examples in this conversational corpus of adult clause chains with different subjects, although these occur in narratives, see examples (6) and (9). In this corpus, the dedicated switch-subject subordinate constructions formed with -*nyangka* was used in all the switch-subject contexts, for instance (32). No examples of the same-subject consecutive action marker -*nytjatjanu* were observed in this sample.


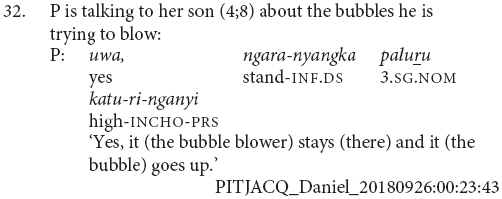


This pattern of usage for clause chain constructions and switch-subject subordinate constructions largely conforms to previous descriptions of the language (e.g., Goddard, 1985; Bowe, 1990). The main difference is that clause chains are less frequent and shorter than suggested by previous descriptions. This is likely, at least in part, due to the wide range of genres and speech contexts contained in the present recordings. Sequences of chained actions are likely to be more common in particular contexts, such as narratives, where speakers are more want to describe sequences of actions (Slobin, 1995).

One construction observed among the adult utterances in this sample is not reported in previous descriptions of Pitjantjatjara. In addition to the clause chains discussed above, there were 13 utterances containing strings of finite verbs in the imperative, (e.g., 33 and 34). These imperative verb strings have no explicit marking of coordination. In contrast to other juxtaposed clauses discussed above, they are impressionistically produced as a single intonational unit and serve the same range of functions (often with the same verbs and in the same contexts) as clause chains. They most typically describe sequences of actions performed by a single actor (e.g., 33 and 34). There is also one example which refers to simultaneous actions, shown here in (35). This strategy has not been discussed in earlier descriptions of the language (e.g., Goddard, 1985; Bowe, 1990; Rose, 2001; Langlois, 2004). All the adult examples are instructions to children and in imperative mood. This construction may be a feature of child-directed speech and potentially restricted to imperatives. Future investigation in a more varied corpus of adult speech would be needed to evaluate this.


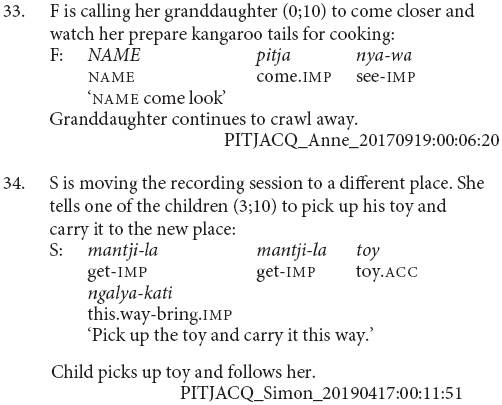



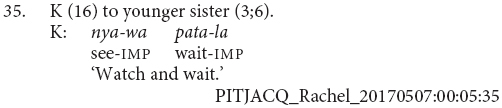


### Stages of Acquisition: Cross-Sectional Analysis

In order to capture the overall progress of acquisition, all utterances from all children with similar MLU (or age, once MLU reached adult levels) were examined to identify patterns of usage. This analysis suggested five clusters, as seen in Table 3.

**TABLE 3:** Children’s clause chain use: number of tokens (percentage of total for that age group).

	Juxtaposed finite		Clause chain			Subordinate	
	Sequential	Simultaneous	Sequential	Simultaneous	Medial only	Switch subject	Same subject
Adults	12 (16%)	1 (1%)	31 (40%)	21 (28%)	4 (5%)	7 (9%)	0
MLU 1.1–1.5	1 (14%)	4 (57%)	0	0	2 (29%)	0	0
MLU 2.1–2.4	3 (60%)	1 (20%)	1 (20%)	0	0	0	0
MLU 2.5–4.1	5 (20%)	1 (4%)	3 (12%)	14 (56%)	2 (8%)	0	0
4 to 6 years	4 (9%)	0	20 (47%)	12 (28%)	6 (14%)	1 (2%)	0
6 to 10 years	3 (21%)	0	5 (36%)	1 (7%)	3 (21%)	2 (14%)	0

A Pearson chi-squared analysis was carried out to test for significant differences in patterns of usage across these initial clusters. This compared proportions of use for each construction type across age groups. Significant differences between groups were noted, χ^2^(1, *N* = 25) = 101.31, *p* < 0.001. *Post hoc* comparisons with Bonferroni correction showed three significantly different groups or stages of acquisition, as discussed below.

#### Stage I: Finite Juxtapositions

In this earliest observed stage of the acquisition of Pitjantjatjara converb constructions, children are typically achieving clause chain functions by juxtaposing finite verbs, rather than using medial verb morphology. This mirrors the earliest stage of Turkish converb acquisition, where children primarily use juxtaposition in place of converb morphology until around the age of 2;6 (Aksu-Koç and Slobin, 1985). In Pitjantjatjara, this pattern was observed among children with MLUs between 1.1 and 2.5 and between the ages of 1;9 and 2;11. See Table 4 for details of individual children within this range.

**TABLE 4:** Clause chain and finite verb juxtapositions produced by children with an MLU between 1.1 and 2.4.

Child	Age	MLU	No. of Utterances	Juxtaposed finite		Clause chain		
				Sequential	Simultaneous	Sequential	Simultaneous	Medial only
A	1;9	1.2	121		1			
S	2;4	1.5	295	1	1			1
S	2;5	1.5	275		2			
F	2;6	2.1	101	1				
D	2;8	N/A	12			1		
E	2;11	2.1	152	1				
W	2;11	2.4	68	1	1			
**Totals**			**1431^1^**	**4**	**5**	**1**	**0**	**1**

*For clarity, zeros are not marked. ^1^Note the total includes all utterances produced by children within this MLU range. Some of these children did not produce relevant utterances during a recording. However, given the low frequency of these constructions and their number of utterances, the absence of tokens could not be taken as evidence that they were not at the same stage of acquisition. Their utterances are, therefore, maintained in the total number of utterances count and used for calculating overall frequency values. Including their utterances yields a frequency of 0.8% for verb juxtapositions plus clause chains, which is the same frequency found when only considering sessions with more than 100 utterances.*

There is a tendency for the younger children, with MLUs between 1.1 and 1.5, to use juxtaposed finite verbs to refer to simultaneous aspects of the same event, as in examples (36) and (37). In contrast, older children, with MLUs between 2.1 and 2.4^7^, tended to use verb sequences to refer to sequential actions, as in examples (38) and (39). This resembles the tendency for English-acquiring children to use multiclausal constructions for describing single before multiple situations (Diessel, 2004). It also bears a resemblance to early word plus gesture combinations, where children initially combine gestures and words in relation to the same element, before combining words and gestures relating to distinct elements, and then finally producing word combinations referring to distinct elements (Kelly, 2014).


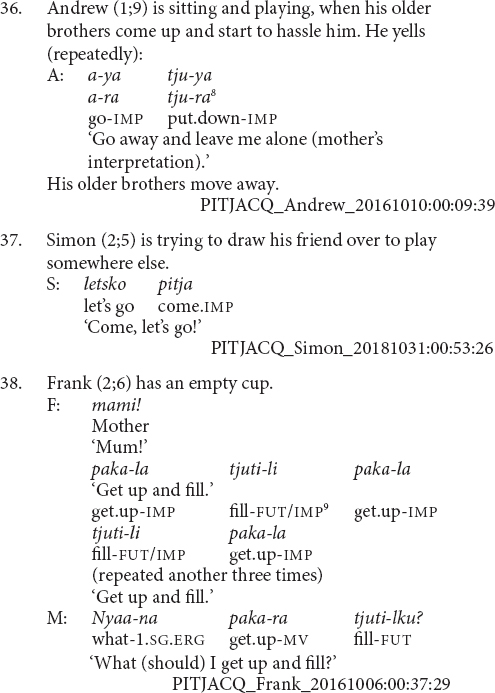



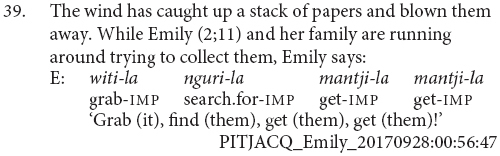


Most of the juxtaposed verbs are in the imperative, as in examples (36, 37 and 39). This is a pattern also observed in the older child and adult finite verb juxtapositions, as in examples (33–35). There is then a question as to whether these juxtapositions are an early form of clause chaining or a specific form of imperative coordination. A definitive answer would require a larger sample, however, the present data suggests these verb juxtapositions are precursors to clause chaining. Firstly, there is a general bias toward the imperative in this dataset. The imperative is the most frequent form of the verb in this dataset: 67% of the verb forms produced by children in this MLU bracket are in the imperative. Imperatives are also the most frequent form of the finite verb within clause chains, making up 50% of all clause chains. Secondly, there are exceptions where other verb forms are juxtaposed. Indeed, only six of the nine verb juxtapositions are imperative, a proportion which perfectly matches that of imperative verb form use more generally within this group. One instance is in the negative and two are in the past. The example in (40) is the clearest with both verbs marked for past tense. The juxtaposition shown in example (41) shows one of the difficulties in judging these cases. Simon is reporting a series of events which occurred in the recent past, so the utterance has a past interpretation. The first verb is clearly marked for past tense. The second verb is pronounced as the bare verb root, which in this conjugation class is the form of the imperative. This second verb could then be interpreted as an (inappropriate) usage of the imperative, a past tense form with the final syllable elided^10^, or a bare verb stem. Finally, these verb juxtapositions are utilized to achieve functions typically performed by clause chains and adult caregivers interpret them as clause chains, as can be seen in (39).


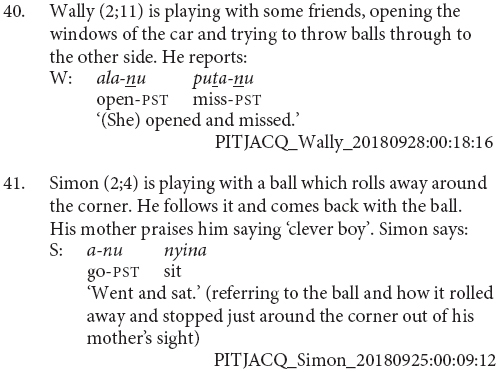


It is interesting to note that while juxtaposed verbs occur in only 1% of the utterances by children within this sample, they appear to be among the early word combinations produced by Pitjantjatjara speaking children. The children with MLUs between 1.1 and 1.5 are all in the two-word stage – where they are producing some two-word utterances among a majority of single word utterances. A count of two-word combination types shows that Action-Action combinations are as frequent as Agent-Negation, Object-Attribute, Possessor-Possessum, and Action-LOCation combinations, as seen in Table 5. Many of these other combination types are typically noted in studies of early word combinations in other languages (Slobin, 1970; Bowerman, 1973), however, I have not seen Action-Action combinations noted. This early combination of actions may be a feature of clause chaining languages.

**TABLE 5:** Two-morpheme utterance types among children with MLUs between 1.1 and 1.5.

Type	Number of tokens	Number of speakers (4 max)	Age range	Example
Action and Agentive	50	3	1;9–2;11	*Mami* (Mummy) *ala* (open)
Action and Objective	24	3	1;9–2;4	*Pala* (that) *nya* (see)
Agentive and Objective	14	2	1;5–2;11	*Mama* (mother) *ama* (breastmilk) [Mother give me breastmilk]
Action and Negation	9	2	1;9–2;5	*Antuntji* (hit) *wiya* (no)
Agentive and Location	9	1	1;11–2;5	*NAME malak* (back) [NAME spray me in the back]
Action and Action	5	2	1;9–2;5	*anu* (went) *nyina* (sit)
Agentive and Negation	5	2	2;1–2;5	*NAME wiya* (NAME, no)
Objective and Attribute	4	1	2;4–2;11	*Pina* (ear) *maru* (dark colored)
Possessor and Possessum	4	2	0;10–2;4	*NAME kuka* (meat) [N’s meat]
Action and Locative	3	1	2;4–2;5	*Paya* (go away) *putu* (far)

Although juxtaposition is the dominant strategy in this age bracket, medial verb forms are not entirely absent. Daniel (2;8) produces the very adult-like clause chain shown in (42). Unfortunately, there are not enough utterances from Daniel at this age to accurately determine his MLU and he has been placed in this bracket based on his age alone. It could be that he is in fact at a more advanced stage of clause chain acquisition. It is also possible that this particular construction is one which Daniel hears more frequently and has thus learnt to repeat.


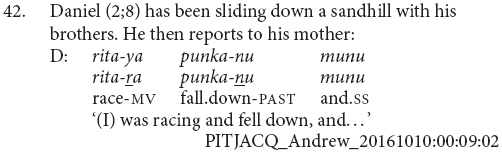


Another use of medial verbs which is more clearly within this bracket is by Simon at 2;4 and 2;5. In each of these sessions, Simon produces nearly 300 utterances with an MLU of 1.5. Across both sessions, he produces four juxtaposed finite verb pairs, mostly in reference to simultaneous aspects. He is, thus, a clear example of Stage I. He also produces a few repetitions of the medial verb form of the verb *pitja* ‘to come,’ *pitja-la*, which he pronounces *pitjaya* due to regular replacement of *l* and *r* by *y.* This sound replacement is well-attested among children acquiring diverse languages (Solé, 2002; McGowan et al., 2004; Klein et al., 2012). There is, however, no discernible difference between his usage of this form and the imperative form *pitja*. This may be an initial step toward the acquisition of the medial verb form.

The tendency toward simultaneous juxtapositions and absence of medial verb usage initially motivated the suggestion of a distinction between children with MLUs between 1.1 and 1.5 from those with more advanced syntax and MLUs between 2.1 and 2.4, as seen in Table 3. However, a *post hoc* chi-squared test showed no significant difference in the use of verb juxtapositions or medial verbs between these two groups, χ^2^(1, *N* = 4) = 7.50, *p* = 0.112. This distinction was, therefore collapsed to yield a single early stage in Pitjantjatjara clause chain acquisition characterized by the juxtaposition of finite verbs. A larger sample would be helpful to determine whether there is indeed a difference between these two groupings within the juxtaposing stage.

#### Stage II: Medial Verbs Are for Modifying

In this stage we see children with MLUs between 2.5 and 4.1 and aged between 2;11 and 3;11 (Table 6). These children are producing adult-like clause chain constructions; however, they are predominately using them for modifying (14 tokens) rather than sequential purposes (3 tokens). This mirrors the tendency for English-acquiring children to use subordinate complex sentences to describe single situations before using them to combine situations (Diessel, 2004). Sequential actions are more commonly described using finite verb juxtapositions (5 tokens) than with clause chains (3 tokens). This pattern of use is significantly different from that of the younger children in Stage I, χ^2^(1, *N* = 4) = 15.07, *p* = 0.005.

**TABLE 6:** Summary of all children in the corpus with an MLU within the range 2.5 and 4.1.

Child	Age	MLU	No. of utterances	Juxtaposed finite		Clause chain		
				Sequential	Simultaneous	Sequential	Simultaneous	Medial only
R	2;11	3.3	110	1		1	4	2
A	3;2	3.2	112	2			1	
A	3;3	3.8	70	2		1	1	
D	3;3	3.2	84				3	
R	3;6	3.2	101			1	1	
T	3;9	4.1	154				2	
D	3;10	2.7	62				1	
E	3;11	2.5	224		1		1	
**Totals**			**964**	**5**	**1**	**3**	**14**	**2**

*For clarity, zeros are not marked.*

It is worth noting the wide MLU range of this stage. For most sessions, the child has an MLU between 3.2 and 3.8. Where the child has a lower MLU, they are mostly conversing with a younger child and are likely modulating their speech accordingly. Both children with lower MLUs are older than most of the children at this stage, aged 3;10 and 3;11, and their use of clause chains is typical of this stage. On the other end of the range, one child, Tabitha (3;9), presents with an MLU of 4.1, which is within the observed adult MLU range. In this session, she is interacting with older children and an adult. She is a noted talker in the community and reported to be advanced for her age by her adult family members, however, her use of clause chains in this recording resembles that of the other 3-year-olds in this stage.

The main characteristic of this stage of acquisition is the use of clause chains for modification rather than sequential actions. Children at this stage appear to use clause chains for the full adult range of modification purposes and there are examples of aspectual (43 and 44), postural (45), and other adverbial uses (46 and 47).


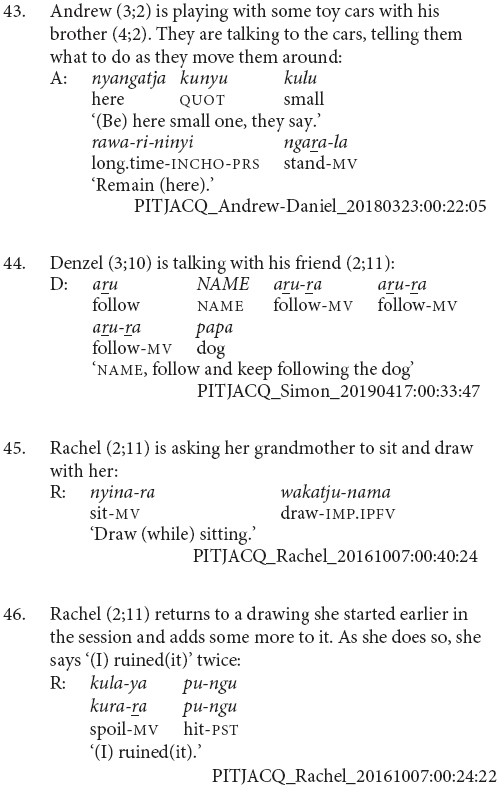



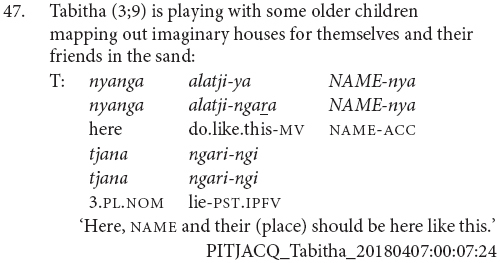


Children at this stage sometimes refer to sequential actions using clause chains, as in examples (48 and 49). However, they more commonly use the juxtaposed finite verb strategy of the previous stage, for instance example (50). There does not appear to be a difference between the children who use each strategy or the particular verb combinations they use them with. It is possible that as children acquire clause chains they are initially specialized for modification as a contrast to the finite verb chain strategy which remains a potential strategy for sequential actions among adult speakers. This would be something to investigate with more fine-grained longitudinal data.


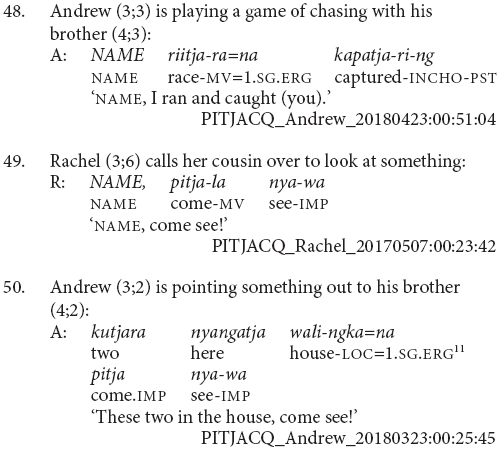


This is also the stage at which children learning Pitjantjatjara start to produce two other complex clause types – purposive and complement clauses. The complement clauses typically function as objects of verb *wangka* ‘say,’ as in (51). The purposive clauses are not yet combined with a finite verb. These uses of purposive clauses independently of a matrix verb, as in (52 and 53), are an acceptable option in adult Pitjantjatjara much like English ‘because it’s raining.’


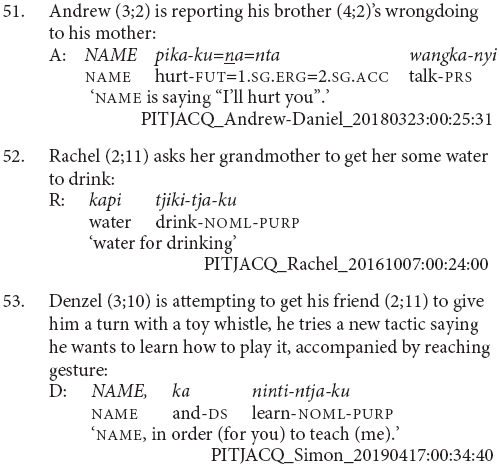


In summary, in this second stage of Pitjantjatjara clause chain acquisition, children regularly use clause chains for simultaneous modifying type functions. They also use clause chains to refer to sequential actions, but still prefer juxtaposed finite verbs for this purpose. The children in this stage are of a similar age to when Turkish speaking children begin to use converb constructions (2;6–3;6, Aksu-Koç and Slobin, 1985). However, Turkish speaking children typically acquire sequential converbs early and only begin to use converbs for other semantic functions such as manner modification around 5 years of age (Çapan, 2013). This may relate to differences between Turkish converbs and Pitjantjatjara clause chains, particularly that Turkish has multiple converb forms with distinct semantic functions, while Pitjantjatjara has a single medial form used for many semantic functions. Pitjantjatjara adults also continue to use juxtaposed finite verb for sequential actions. This means that the strategy initially used by children in both languages is a valid adult strategy in Pitjantjatjara and so may remain a preferred strategy for longer among Pitjantjatjara speaking children. In both languages, converb/clause chain constructions appear to be acquired earlier than other non-finite constructions with noun-like participles, and they may form a link between simple sentences and more opaque non-finite constructions (Aksu-Koç and Slobin, 1985).

#### Stage III: Clause Chains Are for Sequential Actions

This is the final stage of Pitjantjatjara clause chain acquisition observed in this study and includes children aged 4 to 10 years, as seen in Table 7 for individual details. It was initially conservatively divided into two groups: 4- to 6-year-olds and 6- to 10-year-olds, based on the wide age range and the lower number of utterances from 6- to 10-year-olds (414, as opposed to 1160 utterances from 4- to 6-year-olds). *Post hoc* tests, however, showed no difference between the two age groups, χ^2^(1, *N* = 3) = 4.47, *p* = 0.215, and the distinction was collapsed.

**TABLE 7:** Summary of all children in the corpus aged 4 to 10 years.

Child	Age	No. of utterances	Juxtaposed finite		Clause chain			Subordinate
			Seq	Sim	Seq	Sim	Medial only	Switch-subject
W	4;1	123			2	1	2	
D	4;2	160	4		2			
A	4;3	107				2	1	
D	4;3	107			6	2		
D	4;8	268			3	4	2	
D	5;3	143			5	3		1
J	5;9	94			2		1	
A	6;10	45			1	1		
L	7;4	93	1					
S	7;7	85	1		1			
C	9;6	43					1	2
M	9;7	85			2		2	
O	9;9	58	1		1			
**Totals**		**1574**	**7**	**0**	**25**	**13**	**9**	**3**

*For clarity, zeros are not marked.*

This stage is primarily distinguished from Stage II by the use of clause chains as the preferred strategy for describing sequential actions, as in example (54). This is also where children begin to use subordinate switch-subject constructions, as in example (55). These differences between Stage II and Stage III were significant, χ^2^(1, *N* = 5) = 13.83, *p* = 0.017.


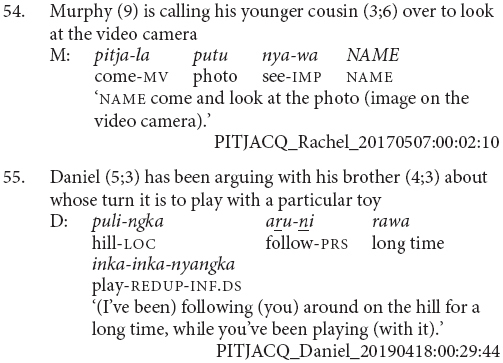


*Post hoc* comparisons with adult usage showed no significant difference, χ^2^(1, *N* = 5) = 5.42, *p* = 0.367. This suggests that Pitjantjatjara children have largely mastered adult-like clause chains and related constructions by around 4 years of age. There is, however, one notable difference which was not included in the statistical comparison and that is the position of the finite verb within the chain. Adult speakers produce the finite verb initially in 21% of clause. In contrast, all of the clause chains produced by 4- to 10-year-olds in this sample were finite-final. In fact, there was only one finite-initial clause chain produced by a child in this corpus and that was the aspect marking ‘remain’ example (43) from Stage II above. This example is also unusual in that the verb *rawaringanyi* ‘do for a long time’ is primarily used as an aspect marker rather than a main lexical verb. There were 38 clause chains produced by children within this stage. Given adult proportions, we would expect 8 of these to be finite-initial. Since none of them were, it is possible that the finite-initial version is acquired later. This connects with the likelihood that the finite-initial chains are syntactically distinct from the finite-final chains discussed above.

It is at this stage that children are first observed using switch-subject constructions in clause chains (56 and 57) and subordinate constructions (55 and 58). Subject changes in clause chains without the use of a different subject marker such as *ka* are not predicted based on previous descriptions of the language (Glass and Hackett, 1970; Goddard, 1985, 1988; Bowe, 1990), but I have observed them occasionally, particularly in child speech.


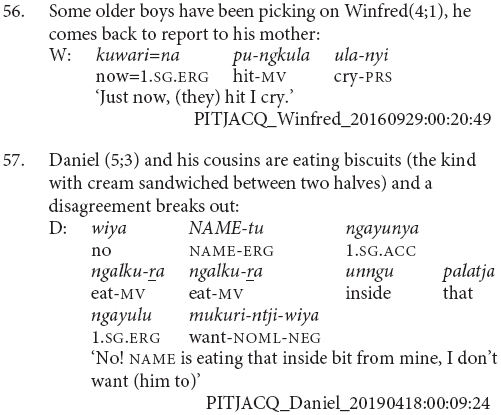



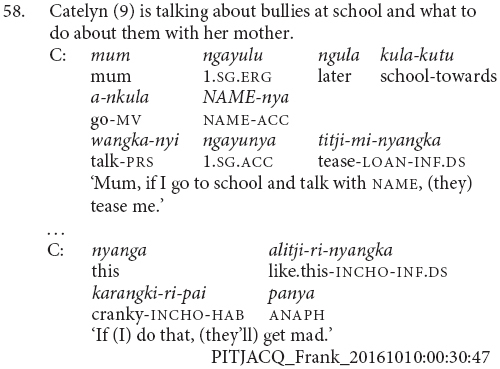


It is also in this stage that we see the first coordinated clauses within a single utterance, by Daniel aged 4;3 (59). This is relatively late compared to English, where children produce clause coordinations from around 2 to 3 years of age (Bloom et al., 1980; Diessel, 2004). This difference is in line with the use of the clause chaining constructions as the standard strategy for expressing sequential actions in Pitjantjatjara.


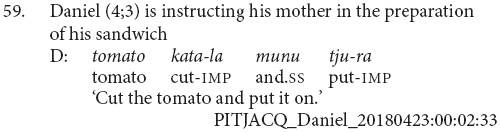


### Longitudinal Progression

Given the structure of the corpus, it is also possible for us to compare these three stages with the longitudinal development of individual children.

Five children were recorded at multiple timepoints within this corpus:

• Simon was recorded on six occasions between the ages of 1;4 and 2;11, however it is only within the recordings at 2;4 and 2;5 that we see clause chainlike constructions. He produces finite verb juxtapositions, largely in reference to simultaneous elements, and isolated medial verb forms, with no discernible difference in meaning from imperative verb forms. This is consistent with Stage I.
• Emily was recorded at 2;11, where she produced one finite verb juxtaposition in reference to sequential actions. This could be consistent with either Stage I or II. She was recorded again at 3;11, where she produced one finite verb juxtaposition and one clause chain, both in reference to postural modifications. This most closely resembles Stage II.
• Rachel was recorded at 2;11, where she produced one finite verb juxtaposition in reference to sequential actions and seven clause chains, only one of which is in reference to sequential actions. This is consistent with Stage II. She was recorded again at 3;6, where she produced two clause chains, one sequential and the other modifying. This is consistent with Stage III but given the small number of tokens could also be consistent with Stage II.
• Andrew first appears in this corpus aged 1;9. During this recording he has an MLU of 1.2 and produces one set of juxtaposed imperative verbs. This is consistent with Stage I. In his recording sessions at ages 2;4 and 2;8, no clause chains or juxtaposed verbs are observed. At age 3;2, he produces juxtaposed finite verbs for sequential action descriptions and a clause chain for aspectual modification. This matches Stage II. At age 3;3, he produces four finite verb juxtapositions and three clause chains to refer to sequential actions as well as one clause chain to refer to manner modification. This could fit with either Stage II or III and may represent a transition between the two. At age 4;3, he produces three clause chains, all for modifying functions. This could match either Stage II or III.
• Daniel first appears in this corpus aged 2;8. During this recording, he produces one clause chain construction to refer to sequential actions. This is atypical of the early stages; however, as we only have 12 utterances from Daniel at this age, it is not possible to draw any clear conclusions. At age 3;3, Daniel is recorded producing three clause chains, all with modifying functions. This is typical of Stage II. At age 4;2, Daniel produces four finite verb juxtapositions, all describing sequential actions, and two clause chains, both for modification. This is again typical of Stage II. At age 4;3, he produces eight clause chains, six for sequential actions and two for modification, as well as one clausal coordination. This is typical of Stage III. At age 4;8, he produces nine clause chains for both sequential and modifying purposes. This is again typical of Stage III. Finally, at age 5;3 he again produces nine clause chains for both sequential and modifying purposes, as well as one switch-subject subordinate construction. This is also typical of Stage III.

The above observations show a progression through each of these three stages of Pitjantjatjara clause chain acquisition, not only when looking across the corpus cross-sectionally, but also when following individual children longitudinally.

### A Summary of Pitjantjatjara Clause Chain Acquisition

The trajectory of clause chain acquisition was divided into three stages. The first stage is characterized by juxtaposition of finite verbs and matches the first stage of Turkish converb acquisition (Aksu-Koç and Slobin, 1985). The second stage is seen with children around 3 to 4 years of age and is characterized by the use of clause chains with medial verb morphology for simultaneous modifying type functions. This differs from the pattern we see with Turkish converbs where children acquire temporal converb functions before other modifying functions (Çapan, 2013). The final stage is observed with children 4 years and older. By this stage, Pitjantjatjara speaking children are also using clause chains for sequential actions and appear adult-like, except for the absence of finite-initial clause chains which are not produced by even the oldest children in this sample.

The majority of chains in this corpus consist of only two verbs. This is true for adults as well as children. There is, however, a significant tendency for chain length to increase with age. This was tested using linear mixed models with the lme4 package (Bates et al., 2018). The dependent variable was chain length with medial-verb only sentences counted as chains of one. Fixed effects were age and type, clause chain or finite juxtaposition. Age was the only significant factor, β = 0.11, *SE* = 0.03, *z* = 3.27, *p* = 0.001.

Overall, clause chains were less frequent in this corpus than predicted based on earlier descriptions of Pitjantjatjara. Clause chains were present in only 3% of adult utterances. Including finite juxtapositions in this brings the overall frequency to 4%. This contrasts with the general ubiquity reported in earlier descriptions of Pitjantjatjara (e.g., Goddard, 1988; Bowe, 1990). I have only observed this ubiquity in written narratives. For instance, a random page of a children’s book is quoted in example (63). Five of the thirteen sentences contain a clause chain, indicated in bold.


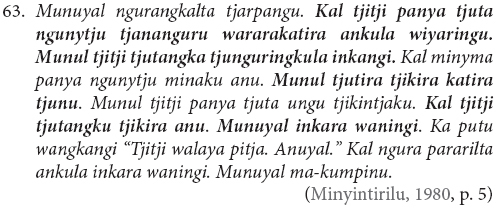


As noted for Turkish (Slobin, 1995), it is likely that clause chains are more frequent in narrative contexts, since they are typically used for event linkage. While there are short spontaneous narratives contained within the present corpus, there are also many other contexts which are less conducive to event linkage. It is possible that previous reports of clause chain ubiquity have been based on speech contexts more consistently conducive to event linkage. Indeed, although clause chaining was less frequent than expected, it was the dominant strategy employed for combining sequential actions. It is also possible that linguists have focused on the possibility of long clause chains, much as there is a focus on long verb complexes in polysynthetic languages although only some slots are in fact generally used in practice (e.g., Ponsonnet, 2015). A more detailed investigation of Pitjantjatjara narratives would be required to resolve this.

## Conclusion

This paper set out to sketch the progress of child acquisition of Pitjantjatjara clause chains. This investigation also uncovered some previously undescribed properties of adult clause chain use. Clause chains are not as frequent in spontaneous speech as previous descriptions have suggested. It is also possible for medial verbs to occur without a finite verb. In these cases they appear to be used for polite imperatives or to mark repeated or continuing actions; note that these functions are also described for medial clauses occurring without a finite clause in Nungon (Sarvasy, 2015). Finally, the dataset showed several cases of finite verb juxtapositions. This is potentially limited to child and child-directed speech.

The investigation identified three stages in the acquisition of Pitjantjatjara clause chains. 1) Pitjantjatjara speaking children begin by juxtaposing verbs without medial verb morphology, to refer to simultaneous aspects of an action or sequential actions. 2) They then use clause chains with adult-like medial verb morphology for modification functions. 3) Finally, they use adult-like (finite-final) clause chains for sequential and modifying functions. The children in the present dataset did not produce adult-like finite-initial clause chains. These may be acquired significantly later.

There are some striking similarities in the acquisition of Turkish and Pitjantjatjara converbs, especially given the differences between the two languages and their social settings. In both languages, children start out producing sequences of finite verbs to achieve clause chain-like functions. This is also a precursor to English coordinated clauses (Diessel, 2004) and the juxtaposed finite verbs of Pitjantjatjara speaking children are potentially a precursor of coordinated clauses and clause chains combining sequential actions.

In Pitjantjatjara, we can see that these early action combinations are among the initial set of word combinations children make before progressing on to longer utterances. Descriptions of two-word combinations in other languages (e.g., Slobin, 1970; Bowerman, 1973) do not list combinations of actions. This may indicate that Pitjantjatjara speaking children combine action elements earlier than children acquiring other languages, possibly due to the influence of clause chains. Alternatively, it may be that researchers have overlooked action combinations as they are not among the standard strategies of the adult language.

In both Pitjantjatjara and Turkish (Aksu-Koç and Slobin, 1985), children are observed to use adult-like clause chain morphology from around the age of 2;6. However, there is a difference in the functions to which they typically apply them. Turkish children typically first use converbs for temporal relations, particularly linking sequential actions. In contrast, Pitjantjatjara speaking children’s early clause chains are typically for modification rather than temporal sequence. This is potentially linked to the greater lexicalization of modificational rather than sequential clause chains in Pitjantjatjara. It is also potentially linked to the fact that the initial finite juxtaposition strategy remains a viable adult strategy for temporal sequences in Pitjantjatjara. It is notable, however, that Pitjantjatjara speaking children appear to employ both finite verb chains and clause chains first for referencing two aspects of a single event and only later in order to join separate elements together. This is potentially linked to Slobin’s (1995) finding that Turkish children do not acquire the converb *-erek* until notably later around the age of 7 years. This converb form is claimed to be used to join two events together into a cohesive unit and it may be that the Pitjantjatjara sequential clause chains have more in common with this Turkish form than the other, earlier acquired, converb forms.

This initial investigation of the acquisition of Pitjantjatjara clause chains provides a foundation for further investigations addressing the manner and timing of Pitjantjatjara clause chain acquisition. It also raises questions regarding the similarities and differences in the acquisition of clause chaining constructions cross-linguistically.

## Data Availability Statement

The datasets generated for this study will not be made publicly available. They contain participants’ personal information and they have chosen not to make it generally available. Requests for more information or to access portions of the data should be directed to the author.

## Ethics Statement

The studies involving human participants were reviewed and approved by the Human Research Ethics Committee of The University of Melbourne (Approval number 1647234). Written informed consent to participate in this study was provided by the participants’ legal guardian/next of kin. Written informed consent was obtained from the individual(s), and minor(s)’ legal guardian/next of kin, for the publication of any potentially identifiable images or data included in this manuscript.

## Author Contributions

The author confirms being the sole contributor of this work and has approved it for publication.

## Conflict of Interest

The author declares that the research was conducted in the absence of any commercial or financial relationships that could be construed as a potential conflict of interest.

## Footnotes

2 The latest census reports 3125 speakers (Australian Bureau of Statistics, 2016). However, census reports can be inaccurate (e.g., Morphy(ed.), 2007; Simpson, 2013; Dixon and Angelo, 2014). My own estimates place speaker numbers at a minimum of 2000.

6 Ages given in year;month format.

7 There were no children in this sample with MLUs between 1.5 and 2.0.

10 Reduction of the final syllable, TAM marker, is common in adult Pitjantjatjara and often only leaves a part of the nasal onset. Syllable deletion is also a common feature of child speech.
